# Narrating the journey of sustained recovery from substance use disorder

**DOI:** 10.1186/s13011-018-0167-0

**Published:** 2018-09-27

**Authors:** Mandy Stokes, Peter Schultz, Assim Alpaslan

**Affiliations:** 1 PO Box 2777, Montana Park, 0159, Pretoria, Gauteng South Africa; 2 University of South Africa, 5th Floor, Room 19, Theo Van Wyk Building, Muckleneuk Campus, Preller Street, Sunnyside, Pretoria, South Africa; 3 University of South Africa, 9th Floor, Room 165, Theo Van Wyk Building, Muckleneuk Campus, Preller Street, Sunnyside, Pretoria, South Africa

**Keywords:** Sustained recovery, Substance use disorder, Support groups, Treatment, Schlossberg’s transition process model

## Abstract

**Background:**

The reported high rate of relapse in the context of an ever-increasing rate of substance abuse internationally and in South Africa together with the fact that the topic of sustained recovery from Substance Use Disorder (SUD) appears to be totally neglected in extant literature and research agendas motivated the researchers to conduct this investigation. The aim was to obtain an in-depth understanding of how individuals recovering from a SUD experience and sustain their recovery in order to fill the gap in the knowledge base.

**Methods:**

A qualitative research approach was followed, employing a narrative and phenomenological research design alongside an explorative, descriptive and contextual strategy of inquiry. Fifteen participants were purposefully recruited; and data were collected by means of individual, face-to-face interviews. Schlossberg’s Transition Process Model (1981) (Schlossberg NK, The Counselling Psychologist 1981;9(2):2-18, Schlossberg NK, Journal of Employment Counselling 2011;48:159-162, Anderson ML et al., Counselling adults in transition: linking

Schlossberg’s theory with practice in a diverse world., 2012) served as a theoretical framework and provided the backdrop to, and foundation for, the presentation of the research findings.

**Results:**

Participants’ entry into recovery was triggered by an internal or external crisis caused by chemical substance abuse. They had to embrace a psychological mind set change, involving commitment to a new way of life in order to sustain their recovery. This, among others, was facilitated by participants’ acceptance of the concept of ‘disease of addiction’ or finding a new faith-based identity. The 12-Step programme and further education and development were found to spiritually support sustained recovery. Strong ongoing support from specialised substance abuse support and/or religious groups, interpersonal relationships with family, spouses and sponsors as well as supportive work environments played a major role in sustaining recovery. The act of helping others further helped the participants to sustain their own recovery.

**Conclusion:**

Regardless of the pathway of recovery, there are key aspects that appear to aid sustained recovery. In grouping these according to the four S’s in Schlossberg’s Transition Process Model (Schlossberg NK, The Counselling Psychologist 1981;9(2):2-18, Journal of Employment Counselling 2011;48:159-162, Anderson ML et al., Counselling adults in transition: linking

Schlossberg’s theory with practice in a diverse world., 2012): self, situation, strategies and support, they seem to facilitate the adaptation to transition from addiction to sobriety. Internal psychological and spiritual resources in terms of self; support (from family, church and support groups); strategies to combat cravings and deal with life problems; and avoiding and managing risk-inducing situations to strengthen recovery.

## Background

Substance use disorder (SUD) refers to a physical, mental and emotional compulsion to use mind and mood altering substances (drugs and alcohol). This compulsion is often accompanied by an increased tolerance to the drug with ever-increasing use; withdrawal when the use of the drug is stopped and continued use despite negative consequences [1–3]. This state of affairs has led to a general perception of society and many professionals and service providers that once you have become addicted, you will always be addicted. This article focuses on participants who have managed to sustain recovery from substance abuse for a period of three years or longer. The probability of continued abstinence increases steeply after three years of abstinence, and longer periods of abstinence are associated with a higher likelihood of continued abstinence [4, 5].

Various definitions of recovery exist amongst professionals and researchers in the field of SUDs as well as persons who are themselves in recovery from substance abuse. The American Society of Addiction Medicine defines recovery as a “state of complete abstinence from all psychoactive substances combined with a satisfactory quality of life” [6]. Everett [7] discusses recovery and concludes that sustained recovery is abstinence from all mind and mood altering substances and sustained changes to life which brings about a sense of balance, control, and consciousness. The Scottish Government defines recovery as a “process through which an individual is enabled to move from his/her problem drug use, towards a drug-free lifestyle as an active and contributing member of society” [8]. It is clear that recovery from SUDs require more than just abstinence but also a focus on developing psychological well-being and quality of life [9]. Cognitive, social and behavioural changes are also noted as essential for recovery in research conducted by Orford et al. [10].

Schlossberg’s Transition Process Model was used as theoretical framework for this study. The Transition Process Model posits that adulthood is a period of change and development characterised by transitions, crises, adaptation, coping and stress [11–13]. A ‘transition’ is regarded as an event or a non-event occurring in a person’s life resulting in belief changes about himself/herself and his/her surroundings, which, in turn, necessitate changes in his/her behaviours and relationships [11–13]. The current study documents the entry of participants into recovery and their adaptation to the transition into sustained recovery from SUD. The model has three major parts, namely approaching transitions: transition identification and the transition process; taking stock of coping resources (Schlossberg’s 4-S system); and taking charge – strengthening resources. The 4-S system divides the individual’s resources into the categories of self (psychological and spiritual resources); support (family and support groups); strategies (coping resources to deal with life problems) and situation (environmental resources) [11–13]. The model developed from the UK Alcohol Treatment Trial (UKATT) identifies a catalyst (trigger) that initiates change alongside treatment and self-initiated commitment which leads to recovery aided by support from family and friends resulting in thinking and acting differently with resultant benefit of positive outcomes [10].

In interpreting the literature consulted [6–8, 11] including Schlossberg’s Transition Model [11–13], the authors’ understood sustained recovery from SUDs as the process of moving from a life of problem substance use towards complete abstinence from all mind and mood altering substances and sustained changes to life which brings about a sense of balance, control, and consciousness [6–8, 11].

Although the topic of substance abuse and SUD treatment, prevention and management has been well-researched internationally [14], there is a lacuna of knowledge focusing on *sustained recovery* from SUDs in the South African and broader African context. This lacuna appears to be caused by a lack of representative epidemiological surveys focusing on rates of recovery and relapse [15, 16]. Treatment in South Africa is regulated by the Prevention and Treatment of Substance Abuse Act (Act no 70 of 2008). It makes provision for governmental, non-governmental and community treatment centres. Most treatment centres are privately owned and are not accessible to the majority of persons caught up in SUDs. Guidance is needed in terms of what it is that helps the person who sustains recovery. The value of this research is to inform and amend current programs in South Africa to make provision for the findings of the research. It was important to select persons from diverse backgrounds (socio-economic and cultural diversity) in the South African context (a country with 11 official languages) and for this reason a qualitative approach was applied in order to gain in-depth knowledge. Kelly et al. [9] concluded in their research in America that certain population groups, for example women, racial/ethnic minority groups and those with opioid and stimulant SUDs require additional assistance to sustain recovery. In South Africa it is the racial majority that cannot access treatment or support groups.

## Methods

The research question, “How do people recovering from a SUD experience and sustain their recovery?” lent itself to a qualitative approach. Qualitative research focuses on meaning and how people interpret the world [17]. The researchers were interested in establishing how participants experienced and made meaning of their experience of sustained recovery. The researchers, furthermore, determined that the qualitative research approach met the goal of the study, which was to obtain an in-depth understanding of how individuals recovering from SUD experience and sustain recovery over a prolonged period.

The narrative and phenomenological qualitative research designs informed the decision and implementation of the data collection method chosen. Narrative research is a qualitative research design that studies the lives of individuals through gathering stories of individuals and retelling these stories [18, 19]. The phenomenological research design involved gathering information from participants to explore their [common] lived experiences about a phenomenon and then analysing and describing the stories to identify the essence of their shared lived experiences [18–20]. In other words, the researchers were interested in the “how is it for you to be in and to sustain your recovery from SUD”; in this way they gave a voice to those who are not often heard in the context of their struggles.

This exploratory and descriptive strategy enabled researchers to gather and describe the information-rich data from participants [21]. The researchers purposely selected a sample of participants from various socio-economic backgrounds, age groups, length of recovery, gender and pathways to recovery in order to ascertain whether, and how, the context in which participants sustained their recovery had an impact on the phenomenon of sustained recovery from SUD.

### Recruitment

Participant recruitment was done by means of purposive sampling through the researchers’ professional (formal) and personal (informal) networks and the internet. The researchers firstly identified the various treatment and post-treatment support structures within the Gauteng area. These included a 12-Step based treatment centre, a faith-based treatment centre, a non-profit community centre, Alcoholics Anonymous fellowship, Narcotics Anonymous fellowship and two faith-based support groups. Gatekeepers from the identified treatment centres and mutual aid support groups were then contacted and meetings arranged to discuss the aim of the research and to obtain the help of the gatekeepers in identifying participants. Inclusion criteria set out at the beginning of the research were to involve participants who were in sustained recovery for three years or longer, over the age of 18, resident in the Gauteng area of Pretoria and Johannesburg, South Africa, and able to speak English. Potential participants were either contacted telephonically or in some instances the field worker attended support groups and presented the prospective research to the group. Participants were given the opportunity to accept or decline participation and those that agreed to participate, signed consent forms. No compensation was awarded/offered to any of the participants.

### Participants

Participants from diverse cultural and social backgrounds, ages, gender and pathways to recovery were recruited in order to obtain perspectives from diverse backgrounds. Participants’ ages ranged from 25 to 78. Nine of the participants were male and six were female. Fourteen participants were employed; and one was retired. Eight of the participants worked in the substance abuse prevention and treatment field. All the participants had been in sustained recovery for at least three years. Twelve participants had three to nine years of sustained recovery; two of them had been in sustained recovery for more than ten years; and one had 41 years of sustained recovery. Eight participants were actively involved in 12-Step programmes; three attended other support groups and the remaining four relied on their work and church environment for support. The participants abused a variety of substances including alcohol, cocaine, heroin, marijuana and khat.

### Data collection

The first author conducted individual, face-to-face in-depth interviews with 15 participants. Interviews lasted an average of 35 min and were conducted in settings convenient to the participants (their home or workplace, or public venue of their choice). All interviews were started with the following opening statement: “Tell me your story of how you managed to sustain recovery over the past few years”. The flexibility inherent in this type of qualitative interviewing allowed the participants to tell their stories from their perspective. Open-ended questions and prompts were strategically used to explore the turning points, support, situation and coping strategies where these were not readily shared by the participants. The researcher used other follow-up probing questions and prompts if and when they were required to explore the depth and breadth of the topic. These questions and prompts included; who and what enabled or supported you to sustain your recovery, what did you find challenging or easy in sustaining your recovery and how has this been helpful for you to sustain your recovery?

With the permission of the participants, interviews were audio recorded. Once data saturation had been reached, the interviews were transcribed.

### Analysis

The first (MS) and third authors (AA) analysed the data independently, using Tesch’s eight steps of data analysis [22–25]. Each interview was read and the underlying meaning was considered. Notes on the content and observations were documented. A list of topics was made and similar topics were grouped together to develop categories. The researchers then met and through a consensus discussion agreed on the categories and themes.

Lincoln and Guba’s model of data verification was used to ensure the trustworthiness of the data obtained [17, 26, 27]. The specific concerns in qualitative research are credibility, transferability, dependability and confirmability.

Credibility is concerned with whether the research findings are an honest and accurate reflection of the participants’ experiences [17, 26, 27]. To confirm the themes that emerged from data analysis the researchers, MS and AA, independently coded the data thereby ensuring triangulation of investigators. The research findings and themes were compared and contrasted to existing literature and Schlossberg’s Transition Process Model [11–13]. Data obtained from the 15 participants were verified and clarified through member checking.

Transferability denotes the degree to which the research findings can be transferred or applied to other contexts, settings, or groups [17, 26, 27]. To allow for a transferability audit by future readers and researchers, a rich, thick description of the setting and participants and a detailed description of the findings of the study are obtainable from the researchers.

Dependability refers to the ability of the study to be replicated in a consistent manner with the same participants in the same context [17, 26, 27]. Strategies to ensure dependability include providing a dense description of the research methods used; triangulation of data sources and investigators and peer examination and consultation [17, 26, 27].

Confirmability, or consistency, is the degree to which the research findings are free from bias; and it occurs when credibility, transferability and dependability have been established [27]. Triangulation of data and theoretical perspectives, ensured confirmability employing independent coding and analysis of the data set by two of the researchers and the use of several theoretical sources to establish neutrality. The researchers engaged in a continuous reflective process throughout the research process, by using a research journal and consulting each other.

## Results

Six main themes emerged through the data analysis process of narratives obtained from the participants.

### Theme 1 - the transitions that put them onto the journey of sustained recovery

In response to the question, “Tell me your story of how you have managed to sustain recovery over the past few years”, it became evident in the stories of five of the participants (presented below) that hitting ‘rock bottom’^1^ or a crisis brought about a turning-point in their lives, putting them onto the road to recovery. These turning points were motivated by internal and/or external motivators [28, 29]. Among the internal motivators, reaching ‘rock bottom’ or ‘burning out’ were mentioned [28, 29]. External motivators mentioned by participants included interventions by relatives or employers.

Participants who experienced internal motivators said the following:

“*What actually happened, my rock bottom happened as it got to the point where there was physical and verbal abuse towards my wife… She had left the home and said that she is leaving me if I didn’t get help and … so I hit a rock bottom…” Male, 47*

“*What brought me to recovery like; I was literally fed up of being fed up. Using* [drugs] *became physically painful, like I had aches in my bones, like my skeleton pained and I was going literally insane. I was seeing stuff, hearing voices and the biggest scare for me was I felt like I was becoming possessed.” Male, 27*

“*I was sitting on my floor; literally I was rock bottom on my floor… I was just miserable. My life was literally just a mess, and I used to weigh 150 kilograms, and I was really depressed, I was ready to commit suicide, you know, with that a lot of abusing alcohol, binging on alcohol.” Female, 29*

“*Yes, so I got to a place where I was suicidal. I was at the windowsill like ready to jump whatever the case may be and my then girlfriend was like ‘what are you doing?’ It was the first time I told her I am trouble, I’m using [CAT, cocaine and alcohol]”. Male, 32*

“*I was at my worst where I just really [changed] from being outgoing and loud at the party, happy. I’d become just like very isolated, withdraw myself, lock myself in my room, aggressive towards my mom and I’ve just got sores over my whole body just knowing that what I am, what I was then is not who I am and I just like really started like praying to God, like there must be something better than this.” Female, 32*

Those who experienced external motivators included a participant who was arrested “*I got arrested* [for possession of drugs] *and then I went to prison for a while and when I came out of prison, I went to rehab in the Karoo.” Male, 33. Another participant was forced by his employer to go to treatment: “They [employer] gave me a choice if I want help or not. So I took the opportunity as I also realised I’ve got a big problem. My wife after 26 years also said she had it, it’s now or never and I went for rehab to [Psychiatric] Clinic.”*

Participants in this research study indicated how they moved out of SUD into recovery. With their movement into recovery the participants had to become familiar with new roles, relationships and routines. Fourteen of the participants reached out for help: nine participants went for in-patient treatment where they were orientated to what recovery entails; four reached out to mutual aid support groups; one asked her church for help and another one was arrested (crisis) and forced to undergo treatment.

“*So I had an amazing six weeks in treatment, my lights all went on, the light bulbs came on, and I absolutely loved rehab.” Male, 47*

“*I went into a treatment centre… and I was faced for the first time with what is actually going on with me.” Male, 27*

“*I got arrested* [for possession of drugs] *and then I went to prison for a while and when I came out of prison, I went to rehab in the Karoo.” Male, 33*

“*I was completely desperate to clean up and fortunately I went to the NA [Narcotics Anonymous] rooms and AA [Alcoholics Anonymous] rooms… I found a sponsor and obviously started working the steps and it continued you know from there.” Male, 42*

“*So I phoned her and I said to her ‘okay I’m ready now,’ and she took me to my first NA meeting.” Female, 28*

“*I went to church and I really just felt like something saying to me if I am going to carry on using drugs I’m going to die. I said to the Pastor that I don’t have a problem, I could still quit whenever I liked because that’s what we always say; we always believe that it’s not a problem. But I said to him I want to see if there’s something better. So I just started getting involved in the church activities but I was still using.” Female, 32*

### Theme 2 - a psychological mind-set as strategy to help sustain their recovery

This theme paid attention to the psychological mind-set that the participants employed as a strategy to help sustain their recovery. The theme was divided into six sub-themes.

#### Conscious decision and commitment to sustained recovery

The participants shared how they had to make a decision to stop using substances (drugs and alcohol) and had to commit themselves to making changes in their lives.

“*It’s about making a deeper than conscious decision that your life needs to change in its entirety. So you will have to let go of everything you were doing. You can’t do the things you were doing and stay clean. You have got to stop doing everything and start doing something else, that’s on the one hand and on the other hand you have to change the way that you look at your life.” Male, 33*

*“… but it is a decision* [referring to sustained recovery] *you need to have to make at the end of the day, to decide you are never going to have a* [another drug again]”.

*“This decision that you’re making, it’s an awesome decision … you’re working a programme, it can’t be wrong* [referring to Step three of the NA programme].” *Female, 42*

#### Accepting addiction as a “disease” or a new faith-based identity

The participants in this study explained how accepting the ‘disease concept’ or having ‘a lifelong illness’ allowed them to start taking responsibility for change.

“*I was faced, for the first time, with what is actually going on with me. That I’ve got the disease of addiction and I could name this dis-ease inside of me that I had all my life and I could start working on it and it was difficult.” Male, 27*

“… *the interesting part to me is the fact that dependency is an illness. It is not just a mind-set and a lot of people especially older people think it’s a mind-set and it’s not, it’s an illness and an illness without a medical cure.” Male, 50*

The participants who followed a faith-based (non-12 step) recovery journey expressed that they do not believe that they have the disease of addiction, but rather that they have been given a new life by God through their faith (a new self-attitude and view of themselves).

“*I personally don’t believe in the ‘once an addict, always an addict.’ I don’t believe in that. I don’t see myself as an addict anymore, because it is not part of my frame of reference anymore. So far back in my past, and I have become a completely new person from the person I used to be then. I see myself as a mother and a wife and a daughter and I work, but that is not … I don’t feel like it is part of me anymore.” Female, 32*

*“I came from a faith based environment where for me the foundation and the new identity* (referring to a non-addict, Christian identity) *that I had received you know from a lot of Biblical input, it helped form an identity for myself that wasn’t, you know, ‘you’re and addict, your past defines you’, you know all that kind of thing. And for me that was quite a revelation because for me that meant that I could change my life without having that label kind of attached to me for ever and a day.” Male, 35*

#### The role of spirituality and religious faith in sustaining recovery

Fourteen of the 15 participants spoke about the importance of coming to believe in something greater than them (a religious entity) regardless of whether they were attending 12-Step meetings, faith-based support groups or no support groups at all.

Some of the participants referred to this as God, others as a Higher Power, or living by spiritual principles. When using their belief in God or a Higher Power as a tool or strategy, participants referred to using prayer, “letting go” and having faith. This belief (faith) became the (meaningful) guidelines for a changed lifestyle of sustained recovery.

*“I also started to put God’s word into action and he says ‘though I walk through the valley at the shadow of death I fear no evil’* [Referring to a passage of scripture from the Bible (New International Version, Psalm 23:4)] *That means actually walking through the valley you know and the things that you fear and the things that you can’t control and I couldn’t control people’s perceptions of me and I had to be honest. Trusting that God would bring me through at the end, at the end of the walk…” (Faith based).*

*“Look, it was, you know I was going to church all the time. But today with a clear mind you know, you can focus a bit more and your faith becomes strong…” (AA).*

*“I started praying every morning… Like I gave to God, praying about it…” (Faith based).*

*“In my recovery, it’s like God played a role, of giving me the strength to be sober, understand? I mean, God is planning everything. God is doing everything. He is helping me figure things out, you know, do this and that.” (State facility).*

*“So, working on my relationship with God is the biggest factor for me to stay clean, because in the past I was immoral, I was everything that God didn’t want me to be and the thing is, like it didn’t bother me because I would use drugs. But now I’m clean, I’ve got a conscience again so I just try and do His will for me. So, that’s the biggest thing that is sustaining my recovery, is to practice God’s will and not my will.” (NA).*

#### The 12-step programme

The eight participants who entered into recovery through the 12-Step fellowships of NA, AA, FAA (Food Addicts Anonymous) and SLAA (Sex and Love Addicts Anonymous) underscored how working the 12-Step programme contributed to their sustained recovery.

“*I’ve tried everything to just change how I perceive myself and like I think through this round of 12-Steps, I’ve learnt to accept myself, I’ve learnt to accept my assets, I’ve learnt to accept my limitations and it’s difficult but my limitations don’t haunt me anymore, they don’t define me because it was like a perfect excuse for me to go act out like…” Male, 27*

“*So that’s how I build my self-esteem, I get to show up for life today. I love life today and these 12-Steps have given me my blueprint through getting a relationship with the God of my understanding…” Male, 32*

#### Personal development and further education

The other participants (not involved in the 12-Step programme) engaged in self-development courses and tertiary education.

“*So to rectify that I went to Mr Google and I started upping my professional skill, my knowledge about my work… I also start using Dr Google and learn about dependency because to help yourself you must know what you are challenging.” Male, 50*

“*You need to enrich your life, you need to carry on, because at times it felt to me like I get stuck with the people here, and I get stuck in the whole thing and working towards something else and becoming something else in your life is also something that kept me sane.” Female, 32*

“*So from there it was really just studying and developing myself so that I could carry on effectively you know sowing into people’s lives… As a coach it took me two years to get a life coaching certification. I am busy doing my international certification now.” Male, 33*

In the process of learning more about themselves, they improved their self-esteem and it provided them with additional coping strategies to deal with stress factors in their lives.

#### Do not forget where you come from

The participants recalled the negative consequences of their addictive lives and shared how the consequences served as reminders and strategy in sustaining recovery.

“*Now, I am still desperate, I tell you. Fear of relapse, fear of losing the little that they have, fear of losing the freedom I found in the fellowship or in the programme… Because when I fall, when I go down, I hit rock bottom times two. No half measures. I don’t take no survivors. It is a complete, complete destruction for me. I can’t stop. So fear in a positive way at the moment. Fear of myself. The desperation is the same.” Male. 42*

“*I was so afraid of going back where I come from, like I was so afraid of the psychosis, I was so afraid of killing myself because of the guilt and shame. I was so afraid of letting down my family. I was so afraid of life, I was so afraid of what I’m going to do next to get drugs… and I’m so fortunate that I’ve stopped when I did, because I don’t know what I would have done next.” Male, 27*

### Theme 3 - social support

Support group attendance and church affiliation played an important role is participants’ journey of recovery.

*“And I just went to meetings* (NA meetings)*, I absolutely just went to meetings” Female, 28*

“*I got introduced, obviously, to the AA. I’d go to the meetings… I enjoyed it…., I survived mainly on meetings – that was my diet. Meetings, and helping other people.” Male, 32*

“*That was it, we went to church on a Sunday and not because we felt we had to, because we wanted to and it was just becoming part of a community of people who lived their lives according to that immovable moral standard and we became part of that …” Male, 33*

The participants also reported close relationships with their spouses and family and newly-established relationships with their sponsors as paramount to their recovery.

“*So the loving and caring support that I got from my grandparents got me through that next thirty days.” Female, 28*

“*So I had amazing family support, my dad as well. For them, like just seeing what the programme did for me, it was such a miracle… So my family is extremely supportive.” Female, 42*

“*And for the first year I spoke to my sponsor every day for fifteen minutes. Every morning I call my sponsor, speak to her for fifteen minutes. And I must say that support structure got me to where I am now. So after the six months, I also started sponsoring, which also contributes to my recovery.” Female, 29*

“*… my sponsor was most fortunately a guy that would always be there as well. He didn’t mind meeting and all of that.” Male, 27*

### Theme 4 - external and environmental changes

Participants stressed the importance of avoiding high risk situations, triggers and staying safe. Strategies to achieve this included keeping busy and being meaningfully engaged to sustain recovery.

*“So I managed to white knuckle it* [figure of speech used in the recovery community to indicate holding on for dear life and resisting using substances] *for that first year, slept a lot, did a lot of meetings, went for coffees, but I didn’t go to any parties, which is where I used to use* [drugs] *a lot.” Female, 28*

*“So, I need to realise like I can’t hang around with criminals, I can’t hang around with still using addicts* [people who are abusing substances]*. I can’t do things that normal people can do and it’s a blessing actually but, so ja, like I said, I don’t blame my community as a hinder to my recovery, but I just feel like to be realistic, like what’s acceptable in my community is not acceptable for me and that’s been a challenge for me to accept.” Male, 27*

“*And at a certain time I picked up painting. Obviously I was a musician and I picked up more instruments. I picked up more books. I picked up other crafts like, leather craft… You know every time I buy tools I get excited, I am going to work on this thing, and I work on it for a month and then the next thing I get bored and then I buy paint and canvas and I will be painting, painting, painting. And next thing then I will buy a new guitar or a saxophone. Or I am stripping it off, which will take me a week to put it back. So for those first five years, life was what I was making.” Male, 42*

*“So my fitness, my health and fitness also gave me an escape. If I was feeling down or I wasn’t having a good day, I loved to go for a jog or a long walk, and that really helped me clear my head.” Female, 28*

“*I started doing things like going swimming…Started running again.” Female, 42*

### Theme 5 - helping others

Twelve of the participants reported the important role helping others or ‘giving back’ formed as part of their journey of recovery. Participants in the 12-Step programmes referred to this as ‘doing service’ [30]. Participants from faith-based treatment and other support group backgrounds also referred to helping others as one of the key factors that helped them sustain recovery.

“*So that’s why we keep being humble, by being of service, and giving of ourselves. ‘Cause AA teaches you, you can’t keep what you got, you got to give it away. You know it’s like a river that’s got to keep flowing, you know. So that’s what we do, you know. You know anybody calls for help here; we don’t hesitate to go and see to them.” Male, 78*

“*So, one of the biggest things I think that helped us [husband and wife] in the beginning was the fact that we were starting a centre and helping other people and I think that is the highest form of purpose because if you can put yourself down to help other people inevitably you are helping yourself actually.” Male, 33*

“*It is genuinely the only way… to help other people to help yourself into the process…. going on a Wednesday night to go talk to people at the rehab centre and try to go and help them cause that is helping them and is helping me and the positive part the feedback that you get there helps you also a lot in improving your self-image to boost yourself and to stay positive in your everyday fight.” Male, 50*

### Theme 6 - work environment

Eight of the 15 participants started working in the addiction recovery field after their recovery, which, in return, supported them to sustain their recovery from SUD. Three participants who are not working in the field of substance abuse treatment also shared that they find a sense of purpose and meaning in their work and a general feeling of being proud of themselves for being able to retain their current work positions.

“*So this is one reason why I am actually clean. It has sustained my recovery learning more about others and myself in the whole system of recovery and I doubt if I wasn’t in the system, the recovery system, maybe I would have picked up. So every day working with addicts has actually helped me.” Male, 47*

“*I’ve always kind of like stayed in the recovery culture. So I’m always around other recovering addicts who speak the same kind of recovery skill language so it’s made it easier. So being in this environment I can see the bad stuff and it reminds me of the bad stuff.” Female, 32*

“*I chose the fitness industry that I’m passionate about where I can give back* [fitting with the principle from the 12-Steps of giving back]*. I can help people feel better about themselves, look better, get fit, get healthy”. Female, 28*

## Discussion and recommendations

The present study examined the personal journeys of 15 South African adults who have managed to sustain recovery from SUDs. Although previous research has investigated and reported on sustained recovery [9, 10, 14] and the recovery capital required to sustain recovery no existing data was found within the South African context. This research confirmed that sustaining recovery in South Africa follows much the same path as it does internationally.

The findings illustrated and confirmed that entry into recovery is triggered by an internal or external crisis caused by substance abuse [10, 11–13, 28, 31]. Various crises, circumstances and reasons were responsible for the participants in this study to initiate their transition and subsequent journey from SUDs to sustained recovery. These turning points were motivated by internal and/or external motivators [28, 29]. Among the internal motivators, reaching ‘rock bottom’ or ‘burning out’ were mentioned [28, 29]. External motivators mentioned by participants included interventions by relatives or employers.

Various pathways to recovery were presented by the participants in the study. The participants reported that they reached out to substance abuse treatment centres; substance abuse mutual aid support groups (such as AA, NA, FAA, Mighty Wings, HEAL and CAD); as well as the church for help once they realised they wanted to move out of SUD into recovery. Regardless of the pathway to the recovery or the help sought out by participants, all the participants involved in the research managed to find a way to sustain their recovery within their financial, religious and socio-economic status and embraced the help that was offered through these different pathways to recovery. These findings are also noted in the literature consulted [32].

The importance of a mind-set change came through amongst participants in 12-Step and faith-based recovery. A commitment to a new way of life was facilitated by participants’ acceptance that they had the ‘disease of addiction’ or a new faith-based identity and the inclusion of spirituality, the 12-Step programme and further education and development to support their sustained recovery. In essence, it is the change in the perception of self (“I am a recovering addict/alcoholic” and “I have a disease”. “I need help to become and remain sober” and “my new identity as result of my faith”) that helped them in their transition. The findings also confirmed that recovery remains an on-going process. Embracing the disease concept of addiction as a lifelong disorder and admitting powerlessness against drugs and alcohol are unique cognitive processes to those who find recovery through the 12–Step disease (Minnesota) model [33, 34]. When linking this subtheme to Schlossberg’s Transition Process Model [11–13], acceptance of the disease concept can be seen as a strategy that modifies the meaning of the problem. Through accepting the disease concept, participants were able to make sense of their past behaviour; and were thereby able to change the meaning they attach to their problem. In addition, this subtheme ties in closely with self in that the participants acquired a more optimistic self-attitude, enabling them to deal with their substance abuse problem once they have accepted that they have a disease which can be treated; and that they have a sense of control over how their lives would proceed in recovery.

A strong theme of the role of spirituality and religion was reported. Spirituality relates to the aspects of the Self and Strategy in Schlossberg’s Transitional Process Model and spirituality has been found to influence coping ability, self-esteem, realisation of personal strengths and resilience [13]. The acceptance of a higher power or God was cited by all the participants. This involved surrendering control over addiction, asking for help and realising that the journey of recovery cannot be taken alone. These reports are universal and supported by existing literature on the importance of spirituality in SUD treatment and sustained recovery [13, 35–37]. The Pathways to Recovery Report [34] reported that 21% of their participants (*n* = 33) said that faith in a higher power was the main path to their recovery; and that they often relied on religious organisations in their communities. While faith is fundamental to faith-based recovery pathways, there are many common spiritual themes in 12-Step pathways as is evidenced in participants’ narratives depicted above.

The process of learning more about themselves improved their self-esteem and provided them with additional coping strategies to deal with stress factors in their lives. The value of the 12-Step programme in sustained recovery from chemical addiction is also confirmed in the literature consulted [38–40], However, the data obtained from the research indicates that the 12-Step programme is not a prerequisite for sustained recovery since participants who entered into treatment and recovery through other pathways, that are not related to a 12-Step programme, also refer to having an orientation to growth and learning and how it contributed to their sustained recovery.

Sustained recovery is enhanced by strong and ongoing support from specialised substance abuse support and/or religious groups. For effective coping during transition, individuals need access to a range of support types. Categories of support according to Schlossberg’s Transition Process model can range from those closest to the individual (interpersonal relationships, such as sponsors and spouses) to those support systems furthest removed (institutions and communities) [11, 13]. Several studies have been conducted in relation to the effectiveness of 12-Step and other support group involvement in sustained abstinence [30, 41, 42]. All confirm the benefits of mutual aid self-help support groups; and all found that continued attendance of mutual aid support groups, whether or not related to the 12-Step programme or other programmes was a strong predictor of a positive outcome; that is, continued abstinence [37, 43–45]. Interpersonal relationships with family, spouses and sponsors play a major role in sustaining recovery [34, 42]. A significant difference that is noted between international and South African support groups is the lack of variety of support groups and limited access to support groups in South Africa. However, participants in this study utilised other resources in terms of self and strategy to sustain their recovery.

Environmental control, such as avoiding high risk people, places and things that might trigger cravings or a desire to relapse, is an essential strategy for maintaining abstinence throughout the recovery journey [39, 40, 46–49]. The literature consulted in this regard correlates with this study’s findings as social networks established during active drug and alcohol use could pose as a threat to recovery [32, 47–51]. Ten of the 15 participants voiced the importance of finding new activities to keep themselves busy in order to combat boredom and find meaning and purpose in their lives.

The act of helping others aids participants in sustaining their own recovery. The act of helping others as a remedy to sustain their recovery from SUD relates to the strategy aspect of Schlossberg’s Transition Process Model [11, 13]. The literature consulted confirmed the benefits of helping others as a strategy to maintain recovery [30, 41, 42]. The benefits of helping others include tranquillity, improved self-worth, greater optimism, raised self-esteem and decreased depression and helplessness [41].

When looking at the concept of work through the lens of Schlossberg’s Transition Process Model, the aspects of self and support come to mind; in terms of the aspect of self, the type of work a person engages have an effect on how a person perceives himself/herself in terms of socioeconomic status, self-efficacy, commitment and spirituality [13]. The work environment in which participants find themselves is supportive of their new lifestyle. The studies by Laudet and White [52] and Duffy and Baldwin [42] confirmed that work, specifically work in the substance abuse treatment field, was high on their participants’ priority lists of what is deemed important in sustaining recovery.

In view of the conclusions depicted above it would be valuable if the research could be replicated on a larger scale to include more participants representing the diverse population of South Africa from all cultural/ethnic groups and 11 official languages as well as different pathways to recovery. In addition, information from this research could be used to assist those entering into recovery to identify and build on their resources.

Given the vast number of persons seeking and entering treatment in South Africa and the reported high relapse rate and unemployment rate, findings of this study of the importance of employment very (Theme 6), it is recommended that research in the form of a pilot project be done which focuses on the value of protective employment and supportive housing, such as that conducted in Blackpool, United Kingdom [53] where participants were involved in building affordable housing for the recovery community.

The psychological and social aspects of what ‘a new self-given by God’ and ‘living according to the gospel’ mean were not explored in depth in this study; and the literature consulted did not provide an adequate explanation of, or focus on, what it means for individuals to enter into recovery and sustain their recovery from a faith-based perspective. This faith-based recovery process warrants further exploration to determine whether these aspects can be transferable to other secular treatment settings.

The incorporation of Schlossberg’s 4-S system focusing on individuals’ resources can improve clinical practice by identifying those resources that are lacking and can be strengthened to ensure sustained recovery. In terms of social work practice and policy it is recommended that a resilience, strength-based perspective be used to assist clients in discovering and strengthening their resources.

Research into the mechanism of the 12-Step programme and how it benefits the development of the competent self in terms of self-efficacy, resilience and hardiness could provide useful data that could be transferable to other non-12-Step treatment options. In addition there is a lack of research in the South African context that focuses on individuals residing in rural areas with limited access to resources, such as treatment centres and mutual aid support groups. Research exploring the needs of these individuals will provide guidelines as to how resources can be built or developed in these areas.

## Conclusion

This study confirmed previous research as to the main aspects necessary to sustain recovery. People can recover from SUDs provided that they are enabled to make sense of the crisis that set them onto the journey of recovery and by making a commitment to a new way of life. This commitment requires a psychological mind-set change involving accepting addiction as a disease which requires lifelong maintenance or incorporating a new faith-based identity as strategy. Commitment also entails taking action and making lifestyle changes that include letting go of old friends and environments and establishing new supportive convoys of support. The adoption of a spiritual or religious way of life is a central theme and linked to this is an altruistic attitude. Having a secure living environment and being financially independent also play significant roles in the ability of people to sustain their recovery. It is hoped that the findings from this research, although anecdotal, will inform policies and legislation in South Africa to further enhance service delivery to those living with SUDs.

### Limitations of the study

Limitations to a research study originates from its conceptual framework and research design [18]. The qualitative nature of this study meant that a small sample size was used owing to time and financial constraints. Small sample size limits the generalisability of the research findings. Recruitment through the gatekeepers could have introduced some potential bias as they could have selected individuals whom they have close contact with and could vouch for their sustained recovery. This led to eight of the participants who were interviewed working in the substance abuse treatment field. However the main aim of the research was to look at sustained recovery and not how different employment affects sustained recovery.

## Footnotes

1 In the context of addiction and recovery ‘rock bottom’ is described as “having had significant losses in one’s life” [50, 54] or “having reached a low point of loss and failure” [51, 55].
